# Bilateral Operation for the Stone, Performed in La Pitié, Hôtel Dieu, and Hospice De Perfectionnement

**Published:** 1828-12

**Authors:** 


### OPERATION FOR THE STONE.

*Bilateral Operation for the Stone, performed in* La Pitié, Hôtel Dieu, *and* Hospice de Perfectionnement. From a Correspondent in Paris.

The operation of Celsus seems to be a favorite with the surgeons of the French metropolis, if we may judge from its performance about the same time at three of the principal hospitals, by M. Lisfranc, M. Dupuytren, and M. Bougon. It consists, as the term implies, in an incision on both sides of the perineum, just above the anus, of a semicircular shape, or nearly so; after which, by careful dissection, the surgeon cuts down upon the groove of the staff into the membranous part of the urethra. The scalpel, or concealed bistoury, completes the incision on both sides of the prostate obliquely downwards. M. Dupuytren uses a double-bladed bistourie caché, which opens so as to effect the incision with the exact degree of obliquity required.

M. Dupuytren's patient, an old man, died after having lingered for two months under symptoms of inflammation in the pelvis, which at first were obscure, and latterly became more marked. To what are we to ascribe the accession of those symptoms which terminated in death? Doubtless to an hemorrhagy that occurred within the bladder and pelvis, in the course of the day after the operation, which nevertheless was performed with dexterity and despatch.

The mode of detecting hemorrhagy within the bladder or the cellular membrane of the pelvis, when no blood passes through the wound, as in the present case, is a point of vast importance; for the life of a patient may be sacrificed to the mere inability of the surgeon to discover the cause of alarming symptoms, and which unfortunately do not manifest themselves until blood has been extravasated in great quantity.

If the effusion be in the bladder, it becomes painful; the patient is seized with shivering, makes continual effort to pass his urine, and accordingly voids filaments of blood through the urethra. Pain is felt in the glans penis, and the distended bladder projects above the pubis. The patient is in continual agitation; is covered with cold sweats; his countenance is pale; his pulse small, frequent, and sometimes scarcely perceptible.

When the blood is only effused within the pelvis, the effort to void the contents of the bladder does not exist, but the other symptoms are the same. On examining, the external parts of the wound will sometimes be found plugged by a coagulum; the perineum will be distended, and of a dark purple colour.

As the practice of keeping the legs close after lithotomy impedes the discharge of blood through the wound in case of hemorrhage, M. D. considers it to be objectionable. External bleeding is easily arrested, but the internal is often detected too late for the salvation of the patient. Moreover, the presence of extravasated blood either in the bladder or in the cellular membrane of the pelvis, may produce fatal inflammation of these parts.

This unfortunate result was witnessed in this patient.

The treatment to be employed under these circumstances must be too obvious to require mention; but it may be proper to add, that, when the blood flows from a point accessible to the eye, M. D. cauterises the vessel with a red-hot iron.

If it has been extravasated within the pelvis, it must be carefully washed away by means of a syringe.

The operation at La Pitié was performed under the superintendence of M. Lisfranc, by one of the pupils. It was for the extraction of a calculus of considerable size, impacted in the neck of the bladder. The division of the prostate became unnecessary in this case. The first bilateral incision having been made, and the stone cut upon, polypus forceps were introduced, by which it was extracted. It was of large size, and found to be articulated at one extremity; a proof that another was left behind. The student accordingly passed his finger into the wound, and, having felt the calculus within the bladder through the distended neck, unavailingly attempted to seize it with the forceps. M. Lisfranc, who stood by, waited several minutes, from a desire to give the young operator the whole glory of bringing his work to a successful termination: he was at length, however, under the necessity of taking the forceps, and, with magical dexterity, extracted a calculus of large dimensions, and of a form which would contribute to impede its exit under the management of less practised hands.

A third portion, of smaller dimensions, then appeared, which was also extracted.

Wherein consisted the difference between the proceedings of the two operators? This was the explanation given; and here I may remark, that it is an invariable practice of the French surgeon freely to unbosom himself to the pupil,+ seizing every circumstance calculated to impart information, and exposing his own errors in thought and deed.

"When," says M. Lisfranc, "I introduced my finger to the calculus, I found the greater part of it situated behind the pubis, and impacted between its branches at the narrowest part of the space between them. To have grasped it by the forceps while in this position, would have been impossible: hence the failure of the first attempt. I saw that the only chance of extracting the stone was to dislodge it from the narrow part of the opening, and to bring it downwards. This I effected with the forefinger; but I had still to contend with another obstacle: it became impacted in a bed of condensed cellular membrane. The introduction of the thumb enabled me to overcome this new difficulty, and instantly to seize the stone with the forceps."

